# QSAR study of phenolic compounds and their anti-DPPH radical activity by discriminant analysis

**DOI:** 10.1038/s41598-022-11925-y

**Published:** 2022-05-12

**Authors:** Ang Lu, Shi-meng Yuan, Huai Xiao, Da-song Yang, Zhi-qiong Ai, Qi-Yan Li, Yu Zhao, Zhuang-zhi Chen, Xiu-mei Wu

**Affiliations:** 1 School of Public Health, Dali University, Dali, Yunnan People’s Republic of China; 2 Yunnan Provincial Key Laboratory of Entomological Biopharmaceutical R&D, Dali University, Dali, Yunnan People’s Republic of China; 3 The First People’s Hospital of Yunnan Province, Affiliated Hospital of Kunming University of Science and Technology, Kunming, Yunnan People’s Republic of China; 4 Yunnan Provincial 2011 Collaborative Innovation Center for Entomoceutics, Dali University, Dali, Yunnan People’s Republic of China; 5 PharmaBlock Sciences (Nanjing), Inc., Nanjing, Jiangsu People’s Republic of China

**Keywords:** Data mining, Statistical methods

## Abstract

Phenolic compounds (PCs) could be applied to reduce reactive oxygen species (ROS) levels, and are used to prevent and treat diseases related to oxidative stress. QSAR study was applied to elucidate the relationship between the molecular descriptors and physicochemical properties of polyphenol analogues and their DPPH radical scavenging capability, to guide the design and discovery of highly-potent antioxidant substances more efficiently. PubMed database was used to collect 99 PCs with antioxidant activity, whereas, 105 negative PCs were found in ChEMBL database; their molecular descriptors were generated with Python's Rdkit package. While the molecular descriptors significantly related to the antioxidant activity of PCs were filtered by *t*-test. The prediction QSAR model was then established by discriminant analysis, and the obtained model was verified by the back-substitution and Leave-One-Out cross-validation methods along with heat map. It was revealed that the anti-DPPH radical activity of PCs was correlated with the drug-likeness and molecular fingerprints, physicochemical, topological, constitutional and electronic property. The established QSAR model could explicitly predict the antioxidant activity of polyphenols, thus were applicable to evaluate the potential of candidates as antioxidants.

## Introduction

Oxidative stress (OS) is the imbalance of redox reactions, which leads to the production of reactive oxygen species (ROS)^1^. The accumulation of ROS in the body or cells causes cytotoxic reaction, thus inducing a variety of pathological injuries, such as cardiovascular diseases, diabetes, tumors and other chronic diseases^2–6^. Therefore, it is feasible to initiate disease treatment with antioxidants to reverse this imbalance by reducing ROS level^7^. In this context, various measures to increase the use of antioxidants have been employed, and source from nature has become an important source of research and development of green and safe antioxidants^5^.

In recent years, a number of nature antioxidant substances, including crude extracts, bioactive partitions, chemical entities, have been discovered from insects^8–12^. As a continuous study of medicinal insects, five new phenolic compounds (PCs) with DPPH radical scavenging activity were isolated from the medicinal insect *Blaps rynchopetera* (Fairmaire), a local Chinese medicine, and their antioxidant activities were equivalent to vitamin C^13^. However, difficulties in separation and enrichment of above-mentioned PCs impeded further development of these lead compounds^14^. The design and synthesis of analogues of PCs from *B. rynchopetera*, would be a feasible alternative to more efficiently and economically prepare antioxidants.

Quantitative structure–activity relationship (QSAR) study is a powerful *in-silico* method in terms of design and discovery of bioactive compounds^15^. The presented work herein aimed to establish predictive QSAR model of PCs and their antioxidant activity with their 2D-structures and physicochemical properties as potential predictors. Multiple linear regression and discriminant analysis are mature multivariate statistical methods with reported application in the establishment of QSAR models^16–19^. Multiple linear regression requires assumptions of homoscedasticity and independent errors, which would likely be violated by data derived from different investigations. Although discriminant analysis could not provide the concrete predicted values of antioxidant effect, it could determine the probability of compounds’ classification without the requirements of modeling assumptions as multiple linear regression does^20,21^. Thus, compounds from different sources could be incorporated together to fit QSAR model. Therefore, discriminant analysis was adopted to establish predictive models of PCs’ molecular characteristics and their antioxidant activity for further molecular design for the discovery and development of efficient antioxidants.

## Data preparation and methods

**Scheme 1:**
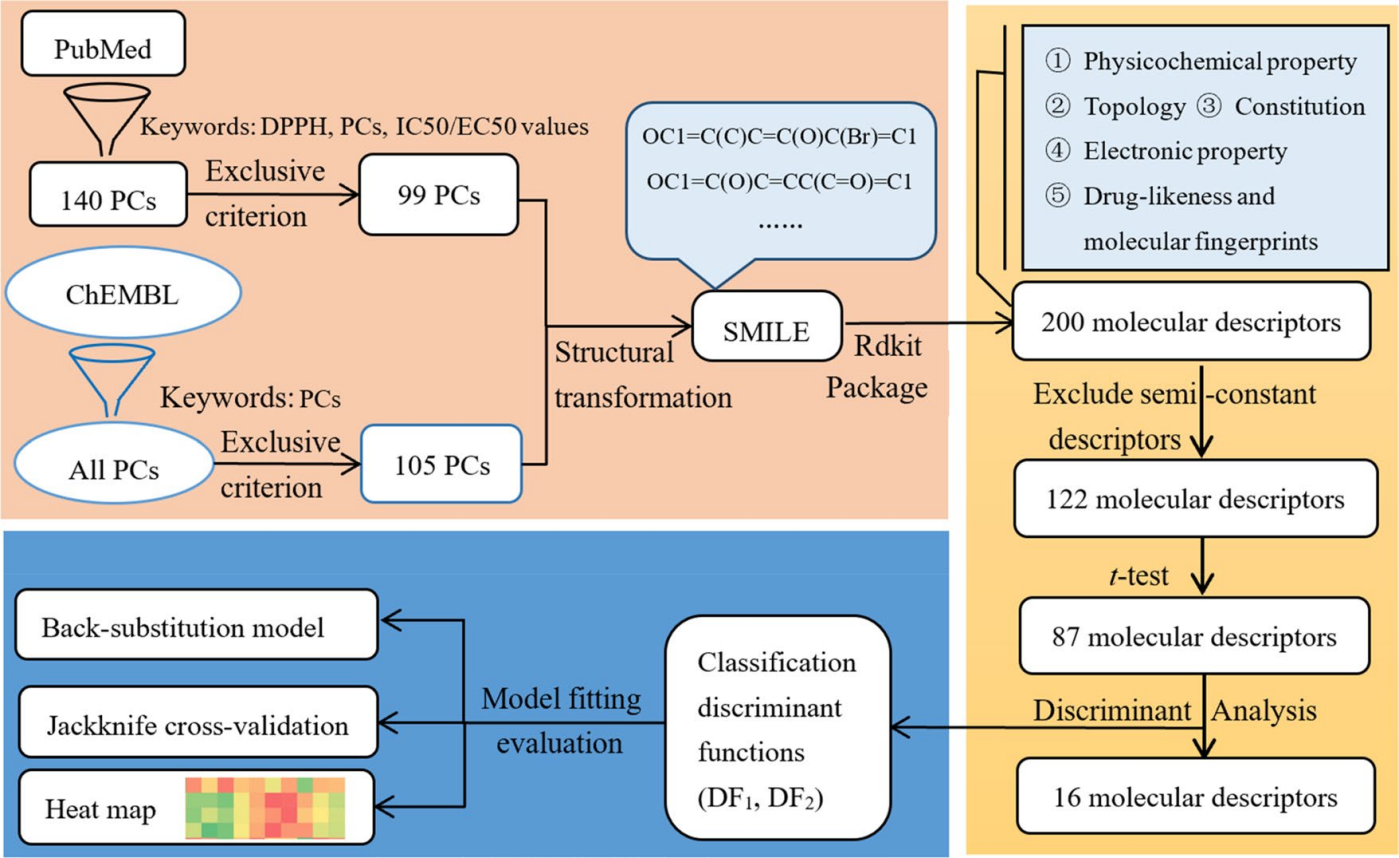
The red area is the process of data mining, yellow is the process of filtering molecular descriptor, and blue is the process of model fitting evaluation.

The strategy of modeling and the selection of molecular descriptors were shown in Scheme 1: (1) Literature retrieval and data collection; (2) Filtering and generating molecular descriptors; (3) Model establishment and fitting evaluation.

### Collection and preparation of compounds

DPPH (Compound **1**) radical scavenging assay is a valid and widely-accepted method for evaluating molecular antioxidant activities. The antioxidant activity was calculated by the rate of DPPH scavenging as Formula (1), and the 50% inhibitory concentration (IC_50_) or 50% effective concentration (EC_50_) values were regarded as indicator for molecular antioxidant activity.

1 $$ {\text{DPPH}}\; {\text{radical}}\; {\text{scavenging}}\; {\text{activity}}\;(\% ) = \frac{{({\text{A}}_{0} - {\text{A}})}}{{{\text{A}}_{0} }} \times 100\% $$

where A_0_ and A are the absorbances in the absence and in the presence of antioxidant, respectively. The absorbance reads for substance could be varied experiment by experiment. Compounds with values of IC_50_/EC_50_ greater than 300 μM are hardly worthy for further development, therefore they would not be treated as lead compounds. Thus, 300 μM was applied as the cut point to differentiate positive and negative samples.

Phenolic compounds (PCs) were derivatives with hydroxy-containing substitutions on aromatic ring of phenol (Compound **2**). The key words of DPPH, phenolic compounds, and IC_50_/EC_50_ values were applied for literature search in the PubMed database. Meanwhile, phenol was used as the keyword to search for negative samples in ChEMBL database.

The overall quality control exclusive criteria for positive samples included the followings: (1) a clear positive control could not be found in the original literature; (2) the IC_50_/EC_50_ values of the same positive control substances from different articles were obviously inconsistent. The exclusive criteria for negative samples were: (1) the IC_50_/EC_50_ values were less than 300 μM in the literature; (2) compound’s DPPH scavenging activity was described though without generalizable IC_50_/EC_50_ values.
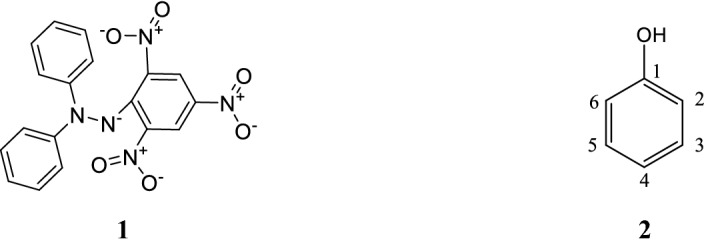


### Determination of molecular descriptors

The 200 molecular descriptors of well-defined, easy-interpretable, and most-often used in QSAR were generated by applying Python's Rdkit package^22^. These molecular descriptors mainly fall into the following categories namely: drug-likeness and molecular fingerprints, physicochemical, topological, constitutional and electronic property^23^. The semi-constant (more than 80% data with the same value) descriptors were excluded to eliminate redundant information and increase power-of-test, consequently, a total of 122 descriptors were retained for later QSAR modeling.

### QSAR model development

The performance of the obtained models mainly depends on their modeling descriptors. The model developed with improper descriptors would lead to either over-fitting or predict weakly and would be useless^23^. The *t*-test was then used to initially filter molecular descriptors that were less relevant with antioxidant activity. The primary objective of discriminant analysis was to build QSAR models that could predict the antioxidant activity of unknown compounds more reliably and precisely. These two statistical analysis methods were performed in SPSS 25.0, and the significance level was set as 0.05.

### Evaluation of model fitting

Two validation techniques along with heat map were applied to assess the accuracy and robustness of the established QSAR model. The following analyses were carried out with SPSS 25.0 and Microsoft® Excel® 2019.

1. *Back-substitution method* By comparing the predicted classification of the discriminant function and the actual classification, the correct discriminant proportion of the classification function was calculated.
2. *Jackknife (Leave-One-Out cross-validation)* (i) Individual sample was sequentially treated as predicative subset, and established the discriminant function with the remaining *N*-1 samples as training subset; (ii) Judging the correctness of predictive classification of predicative subset; (iii) Repeat the above two steps *N* times; (iv) Generating the correct classification proportion.

The error discriminant proportion (Formula (2)) of 10% or 20% was most often used as the standard to evaluate the established model, that is, those error discriminant proportion in validation check less than the standard suggestion satisfactory established model^20^. Furthermore, Kappa consistency coefficients (Formula (3)) were employed to evaluate the stability of the model.

2 $$ {\text{Error}} \;{\text{discriminant}} \;{\text{proportion}} = \frac{{{\text{PN}}}}{{{\text{AP}}}} \times 100\% $$

where, PN is the number of predicted negative samples, and AP is the number of actual positive samples.

3 $$ {\text{Kappa}} \;{\text{consisitency }}\;{\text{coefficient}} = \frac{{{\text{P}}_{0} - {\text{P}}_{{\text{e}}} }}{{1 - {\text{P}}_{{\text{e}}} }} $$

where, P_0_ is observed coincidence rate, and P_e_ is chance coincidence rate.

(3) The performance of established discriminant models on the randomly selected samples consisted each of 10 positive and 10 negative samples respectively were visually presented by heat map.

## Results

### Structural molecular data

In total 140 PCs with unambiguous DPPH radical scavenging activity were collected from PubMed database, after sifting through chemicals and rearranging duplicated labels for compounds with the same structure, 99 PCs were eventually included. Meanwhile, keeping phenol as the search keyword, negative samples in ChEMBL database, with a total of 8721 compounds were presented. To make the sample size of negative subset comparable to positive one, after sifting through the first 1200 pieces of data through exclusion criteria, 105 PCs were finally selected as negative subset (Scheme 1). The chemical structures, IC_50_ values and literature source of 99 positive samples, whereas the chemical structures of 105 negative PCs were available in the supplemental document.

### Initially filtered arguments

The *t*-test was performed to opt 87 molecular descriptors significantly related to the antioxidant activity of PCs (*P* < 0.05) (Table 1). Among them, 4 molecular descriptors belonged to drug-likeness and molecular fingerprints, whereas 35, 5, 13 and 30 molecular descriptors belonged to physicochemical property, electronic property, topological property, and constitutional property, respectively.

**Table 1:** Results of filtering molecular descriptors by *t*-test.

Descriptors	*t*	Type^a^	Descriptors	*t*	Type^a^	Descriptors	*t*	Type^a^
qed	− 10.570	DF	PEOE_VSA14	4.316	P	HeavyAtomCount	4.415	C
MinAbsPartialCharge	8.807	E	PEOE_VSA2	6.152	P	NHOHCount	6.640	C
FpDensityMorgan2	− 4.704	DF	PEOE_VSA3	2.684	P	NOCount	6.429	C
FpDensityMorgan3	− 4.755	DF	PEOE_VSA6	− 6.030	P	NumAromaticCarbocycles	4.053	C
FpDensityMorgan1	− 4.376	DF	PEOE_VSA8	2.058	P	NumAromaticRings	3.794	C
HeavyAtomMolWt	4.695	P	PEOE_VSA9	2.629	P	NumHAcceptors	6.024	C
MaxAbsEStateIndex	6.917	T	SMR_VSA1	7.549	P	NumHDonors	7.201	C
MaxAbsPartialCharge	− 7.485	E	SMR_VSA10	5.525	P	NumHeteroatoms	6.315	C
MaxEStateIndex	6.917	T	SMR_VSA3	− 4.382	P	NumSaturatedHeterocycles	− 2.706	C
MinPartialCharge	7.485	E	SMR_VSA4	− 2.525	P	NumSaturatedRings	− 2.230	C
ExactMolWt	4.331	P	SMR_VSA5	− 2.577	P	MolMR	2.875	P
MolWt	4.330	P	SMR_VSA6	− 5.399	P	fr_Al_COO	3.677	C
NumValenceElectrons	3.988	E	SMR_VSA9	9.010	P	fr_Ar_OH	10.104	C
MinEStateIndex	− 7.975	T	SlogP_VSA11	8.878	P	fr_COO	3.849	C
MinAbsEStateIndex	− 10.317	T	SlogP_VSA2	2.459	P	fr_COO2	3.849	C
MaxPartialCharge	8.807	E	SlogP_VSA3	2.541	P	fr_C_O	8.317	C
BertzCT	6.238	C	SlogP_VSA5	− 2.360	P	fr_C_O_noCOO	7.579	C
Chi0	4.749	P	SlogP_VSA8	4.451	P	fr_NH0	− 5.262	C
Chi0n	2.881	P	TPSA	7.602	P	fr_Ndealkylation2	− 4.482	C
Chi0v	2.792	P	EState_VSA1	3.270	T	fr_aldehyde	3.849	C
Chi1	4.148	P	EState_VSA10	6.841	T	fr_allylic_oxid	4.613	C
HallKierAlpha	− 9.187	P	EState_VSA2	12.192	T	fr_aryl_methyl	− 2.603	C
Kappa1	3.852	P	EState_VSA3	3.701	T	fr_benzene	4.053	C
Kappa2	2.233	P	EState_VSA4	− 5.536	T	fr_bicyclic	2.508	C
Kappa3	2.431	P	EState_VSA7	− 2.701	T	fr_ester	2.899	C
LabuteASA	3.827	P	EState_VSA8	− 6.401	T	fr_ether	2.749	C
PEOE_VSA1	5.858	P	EState_VSA9	4.202	T	fr_phenol	10.104	C
PEOE_VSA11	9.775	P	VSA_EState9	6.962	T	fr_phenol_noOrthoHbond	9.761	C
PEOE_VSA12	3.818	P	FractionCSP3	− 8.698	C	fr_piperdine	− 3.833	C

All the *P* values were less than 0.05.

^a^The types of molecular descriptors: “DF” denotes drug-likeness and molecular fingerprints “P” denotes physicochemical property, “T” denotes topological property, “C” denotes constitutional property, “E” denotes electronic property.

### Establishment of QSAR model

Discriminant analysis was applied to establish QSAR model by incorporating above-mentioned 87 molecular descriptors. Estate_VSA2 and FractionCSP3 were first two included in Model 1 and 2 (Step 1 and 2 in stepwise discriminant analysis), but removed from final model (Final model was obtained in 20th step.). The 16 descriptors included in the final model (*P* < 0.001) were listed in Table 2 with the sequency of inclusion of model fitting.

**Table 2:** Results of discriminant analysis.

Independent Variable	Molecular descriptors	Lambda	*P*	Coefficients of positive samples	Coefficients of negative samples
X_1_	qed	0.408	< 0.001	47.691	62.455
X_2_	MinAbsPartialCharge	0.337	< 0.001	19.218	− 52.885
X_3_	FpDensityMorgan2	0.177	< 0.001	− 67.637	− 112.116
X_4_	FpDensityMorgan3	0.172	< 0.001	14.345	30.362
X_5_	FpDensityMorgan1	0.239	< 0.001	151.179	200.918
X_6_	MinAbsEStateIndex	0.337	< 0.001	23.987	43.389
X_7_	Kappa2	0.303	< 0.001	0.353	2.401
X_8_	PEOE_VSA6	0.229	< 0.001	0.416	0.820
X_9_	SMR_VSA4	0.258	< 0.001	− 0.458	− 0.078
X_10_	SlogP_VSA5	0.182	< 0.001	− 0.481	− 0.614
X_11_	VSA_EState9	0.196	< 0.001	2.353	1.871
X_12_	NOCount	0.211	< 0.001	− 9.660	− 5.499
X_13_	fr_C_O_noCOO	0.248	< 0.001	− 10.962	− 14.661
X_14_	fr_allylic_oxid	0.380	< 0.001	− 2.070	− 3.966
X_15_	fr_aryl_methyl	0.167	< 0.001	2.719	3.980
X_16_	fr_ester	0.275	< 0.001	− 7.376	-0.809

Combined Tables 1 and 2, it could be found that in the final QSAR model, the molecular descriptor of drug-likeness and molecular fingerprints included qed (X_1_), FpDensityMorgan2 (X_3_), FpDensityMorgan3 (X_4_) and FpDensityMorgan1 (X_5_); those of physicochemical property contained Kappa2 (X_7_), PEOE_VSA6 (X_8_), SMR_VSA4 (X_9_) and SlogP_VSA5 (X_10_); those of topological property comprised of MinAbsEStateIndex (X_6_) and VSA_EState9 (X_11_); those of constitutional property consisted of NOCount (X_12_), fr_C_O_noCOO (X_13_), fr_allylic_oxid (X_14_), fr_aryl_methyl (X_15_) and fr_ester (X_16_); and the electronic property was represented by MinAbsPartialCharge (X_2_). The classification discriminant functions (DF_1_, DF_2_) were therefore generated based on estimation of corresponding *β* values (Table 2).

$$ {\text{P}}\;\left( {{\text{y}} = {1}|{\text{x}}} \right) = \left( { - {88}.{15}0} \right) + {47}.{691} \times {\text{X}}_{{1}} + {19}.{218} \times {\text{X}}_{{2}} + \left( { - {67}.{637}} \right) \times {\text{X}}_{{3}} + {14}.{345} \times {\text{X}}_{{4}} + {151}.{179} \times {\text{X}}_{{5}} + {23}.{987} \times {\text{X}}_{{6}} + 0.{353} \times {\text{X}}_{{7}} + 0.{416} \times {\text{X}}_{{8}} + \left( { - 0.{458}} \right) \times {\text{X}}_{{9}} + \left( { - 0.{481}} \right) \times {\text{X}}_{{{1}0}} + {2}.{353} \times {\text{X}}_{{{11}}} + \left( { - {9}.{66}0} \right) \times {\text{X}}_{{{12}}} + \left( { - {1}0.{962}} \right) \times {\text{X}}_{{{13}}} + \left( { - {2}.0{7}0} \right) \times {\text{X}}_{{{14}}} + {2}.{719} \times {\text{X}}_{{{15}}} + \left( { - {7}.{376}} \right) \times {\text{X}}_{{{16}}} \;\left( {{\text{DF}}_{{1}} } \right) $$

$$ {\text{P}}\;\left( {{\text{y}} = {2}|{\text{x}}} \right) = \left( { - {112}.{8}0{7}} \right) + {62}.{455} \times {\text{X}}_{{1}} + \left( { - {52}.{885}} \right) \times {\text{X}}_{{2}} + \left( { - {112}.{116}} \right) \times {\text{X}}_{{3}} + {3}0.{362} \times {\text{X}}_{{4}} + {2}00.{918} \times {\text{X}}_{{5}} + {43}.{389} \times {\text{X}}_{{6}} + {2}.{4}0{1} \times {\text{X}}_{{7}} + 0.{82}0 \times {\text{X}}_{{8}} + \left( { - 0.0{78}} \right) \times {\text{X}}_{{9}} + \left( { - 0.{614}} \right) \times {\text{X}}_{{{1}0}} + {1}.{871} \times {\text{X}}_{{{11}}} + \left( { - {5}.{499}} \right) \times {\text{X}}_{{{12}}} + \left( { - {14}.{661}} \right) \times {\text{X}}_{{{13}}} + \left( { - {3}.{966}} \right) \times {\text{X}}_{{{14}}} + {3}.{98}0 \times {\text{X}}_{{{15}}} + \left( { - 0.{8}0{9}} \right) \times {\text{X}}_{{{16}}} \;\left( {{\text{DF}}_{{2}} } \right) $$

where, y = 1 means the belongingness of positive subset, y = 2 means the belongingness of negative subset.

**Figure 1:**
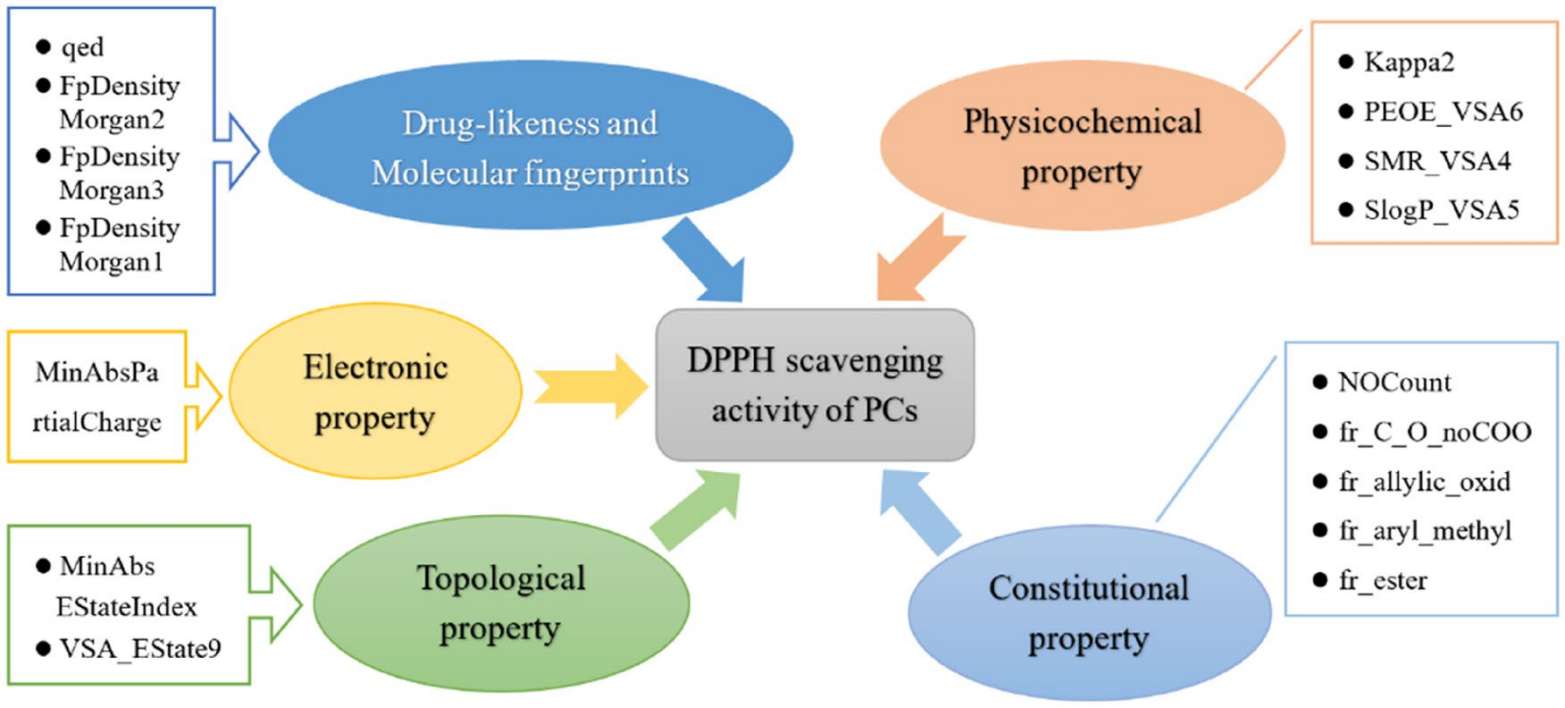
Relationship between antioxidant activity and molecular descriptors of PCs.

In addition, Fig. 1 illustrated the correlation between molecular descriptors and DPPH radical scavenging activity of phenolic compounds. It was revealed that the antioxidant activity of PCs against DPPH was related to all types of molecular descriptors including drug-likeness and molecular fingerprints, physicochemical, topological, constitutional and electronic property.

### Evaluation of model fitting

As shown in Table 3, the classification results of back-substitution method were as following: 99 cases were positive samples, and 96 cases were determined as positive subset, with the correct discrimination proportion of 96.97%; As for the negative subset, all of the 105 chemical identities were determined negative, with the correct discrimination proportion of 100.00%. Furthermore, the Kappa consistency coefficient of 0.971 suggested high consistence between the predictive classification and actual classification. Jackknife cross-validation was employed to further evaluate the stability of the discriminant functions. The correct discriminant proportions were 95.96% and 99.05% for positive and negative samples, respectively (Table 4). The Kappa consistency coefficient calculated based on jackknife results was 0.951.

**Table 3:** Classification results of back-substitution method.

Predicted group membership	Actual classification		Total
	Positive samples (%)	Negative sample (%)	
Positive samples	96 (96.97%)	0 (0%)	96
Negative sample	3 (3.03%)	105 (100.00%)	108
Total	99	105	204

^.^McNemar’s test: *P* = 0.250.

**Table 4:** Classification results of jackknife cross-validation method.

Predicted group membership	Actual classification		Total
	Positive samples (%)	Negative sample (%)	
Positive samples	95 (95.96%)	1 (0.95%)	96
Negative sample	4 (4.04%)	104 (99.05%)	108
Total	99	105	204

McNemar’s test: *P* = 0.375.

**Figure 2:**
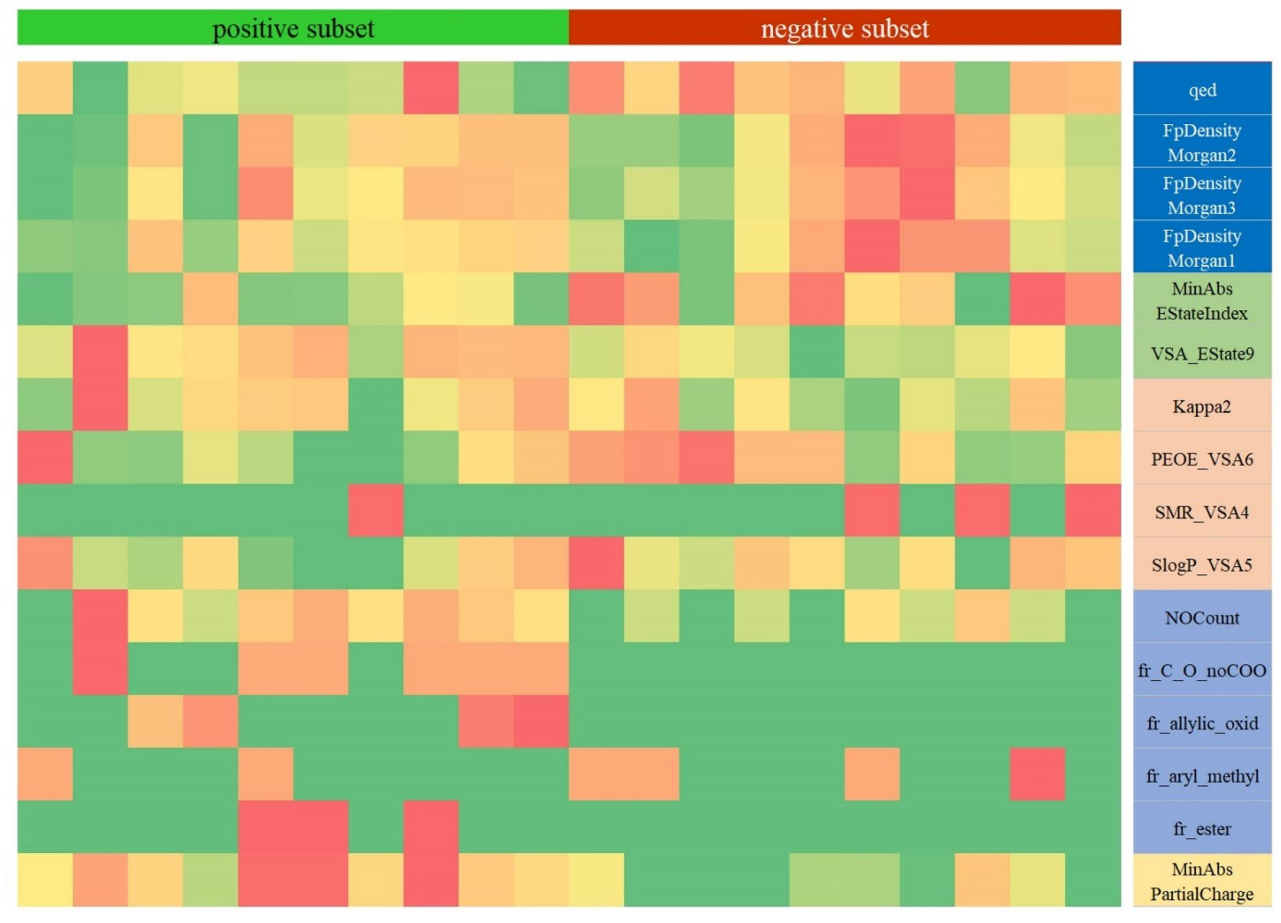
Heat map of the molecular descriptors matrix.

The results of both validation methods supported the accuracy and robustness of the obtained QSAR model. The heat map further visually validated the established QSAR model. As shown in Fig. 2, the color of the first and third molecular descriptors of the positive samples basically tend to be green, while the color of the negative samples tend to be red. The color of the two kinds of samples was opposite to the former on the fourth and fifth kinds of descriptors. Whereas the second kind of descriptor could distinguish the two kinds of samples effectively.

## Discussion

Positive samples were composed of PCs with definite DPPH radical scavenging activity, so it was easier to collect chemical structures with specific IC_50_/EC_50_ values in PubMed database. However, it was not applicable to search PCs without anti-DPPH radical activity. Therefore, the strategy of data mining to establish training sets was designed as the positive samples searched in PubMed database and negative samples collected in ChEMBL database.

In addition to that the assumption of homoscedasticity and uncorrelated residuals might be violated in positive subset, the negative subset could not be included in modeling fitting of multiple linear regression due to the lack of concrete IC_50_/EC_50_ values. Therefore, stepwise linear discriminant analysis was employed to establish more robust QSAR model. In the point of view of drug discovery and development, the potential of molecular druggability demonstrated by positive bioactivity is more important than predicting their IC_50_/EC_50_ values.

According to the molecular descriptors in the final model, each single type of chemical descriptors was indispensable to predict the DPPH scavenging activity of PCs, since at least one descriptor was included for corresponding type. The application of bond dissociation energy (BDE) on the antioxidant of compounds based on Gaussian methods was extensively reported^25–27^. However, inconsistent results might be found for the same compound when using different algorithm, which impeded the generalization of BDE in terms of the molecular antioxidant prediction. In addition, we had attempted to establish QSAR models with the physicochemical properties and/or electronic properties of studied compounds as independent variables, but failed in obtaining a robust and reliable model. The success of drug development not only depend on molecular bioactivity. The toxicity and pharmacokinetics of the hits also play important role and should be considered^24^. The drug-likeness and molecular fingerprints could well reflect certain similarities of drug in constitutional and physicochemical properties of related to compounds’ absorption, distribution, metabolism, excretion or toxicity (ADMET) properties. Compared to other bioactivity prediction model with single independent variable like BDE and our previous modeling experiments, it could be reasonably projected that establishment of the discriminant functions was based on the RDkit integrated molecular descriptors and possessed more accurate and robust prediction since the valuation of molecular bioactivities have been fully explained by the 5 types of chemical descriptors. Furthermore, the QSAR model established based on the discriminant function could predict the antioxidant activity of compounds relatively effectively, but it could not be judged chemical mechanism of antioxidant of individual compound. However, since the included descriptors contain 5 aspects of indicators, therefore, it could be reasonably assumed that the phenolic compounds with positive antioxidant activity display their bioactivity based on various mechanisms.

The error discriminant proportions found in both back-substitution method (0% and 3.03%, Table 3) and Leave-One-Out cross-validation method (4.04% and 0.95%, Table 4) were all less than 10%, hence the goodness of fit of the obtained QSAR model was satisfactory. According to the results shown in Table 3, the validity of established QSAR model was outstanding with Youden index close to 1 (Sensitivity = 0.9697, Specificity = 1).

Heat map, a vivid visualization method, was employed to qualitatively and objectively reflect the correlation between inclusive molecular descriptors and corresponding chemicals. Figure 2 illustrated the adequateness of the established QSAR model by manifestly different color patterns between the positive and negative samples.

The presented discriminant equations could provide in silico screening for the DPPH scavenging activity of PCs with new structures prior to in vitro and in vivo bioassays, in turn to form more economic and efficient drug development protocol. Inevitably, the study results confined the application to DPPH scavenging activity. To explore molecular antioxidant activities of other types such as superoxide anion radical scavenging activity and ferric ion reducing antioxidant power, it calls for additional investigations based on the same strategy as presented.

## Conclusions

The DPPH radical scavenging activity of PCs was related with 5 types of chemical descriptors namely drug-likeness and molecular fingerprints, physicochemical, topological, constitutional and electronic property. The reported model could serve as templates of QSAR for various parent nuclear compounds with different bioactivities.

## Supplementary Information

Supplementary Information.

## Data Availability

All data generated or analysed during this study are included in this published article [and its supplementary information files].

## References

[1] Cabello-Verrugio C, Simon F, Trollet C, Santibañez JF (2016). Oxidative stress in disease and aging: Mechanisms and therapies. Oxid. Med. Cell. Longev..

[2] Lee P, Chandel NS, Simon MC (2020). Cellular adaptation to hypoxia through hypoxia inducible factors and beyond. Nat. Rev. Mol. Cell Biol..

[3] Forman HJ, Davies KJA, Ursini F (2014). How do nutritional antioxidants really work: Nucleophilic tone and para-hormesis versus free radical scavenging in vivo. Free Radic. Biol. Med..

[4] Vendrov AE, Vendrov KC, Smith A, Yuan J, Sumida A, Robidoux J, Runge MS, Madamanchi NR (2015). NOX4 NADPH oxidase-dependent mitochondrial oxidative stress in aging-associated cardiovascular disease. Antioxid. Redox Signal..

[5] Guerra-Araiza C, Álvarez-Mejía AL, Sánchez-Torres S, Farfan-García E, Mondragón-Lozano R, Pinto-Almazán R, Salgado-Ceballos H (2013). Effect of natural exogenous antioxidants on aging and on neurodegenerative diseases. Free Radic. Res..

[6] Pickering RJ, Rosado CJ, Sharma A, Buksh S, Tate M, de Haan JB (2018). Recent novel approaches to limit oxidative stress and inflammation in diabetic complications. Clin. Transl. Immunol..

[7] Mitra I, Saha A, Roy K (2012). Development of multiple QSAR models for consensus predictions and unified mechanistic interpretations of the free-radical scavenging activities of chromone derivatives. J. Mol. Model..

[8] Kim J, Kim K, Yu B (2018). Optimization of antioxidant and skin-whitening compounds extraction condition from tenebrio molitor larvae (Mealworm). Molecules.

[9] Pyo S, Kang D, Jung C, Sohn H (2020). Anti-thrombotic, anti-oxidant and haemolysis activities of six edible insect species. Foods.

[10] Dutta P, Dey T, Manna P, Kalita J (2016). Antioxidant potential of *Vespa affinis* L., a traditional edible insect species of North East India. PLoS ONE.

[11] Park HG, Lee KS, Kim BY, Yoon HJ, Choi YS, Lee KY, Wan H, Li J, Jin BR (2018). Honeybee (*Apis cerana*) vitellogenin acts as an antimicrobial and antioxidant agent in the body and venom. Dev. Comp. Immunol..

[12] Sarasa Bharati A, Ali M (2011). Effect of crude extract of Bombyx mori coccoons in hyperlipidemia and atherosclerosis. J. Ayurveda Integr. Med..

[13] Xiao H, Yin T, Dong J, Wu X, Luo Q, Luo J, Cai L, Ding Z (2017). Five New phenolic compounds with antioxidant activities from the medicinal insect blaps rynchopetera. Molecules.

[14] Zhang Y, Luo J, Han C, Xu J, Kong L (2015). Bioassay-guided preparative separation of angiotensin-converting enzyme inhibitory C-flavone glycosides from *Desmodium styracifolium* by recycling complexation high-speed counter-current chromatography. J. Pharm. Biomed. Anal..

[15] Harsa AM, Harsa TE, Diudea MV (2016). QSAR and docking studies of anthraquinone derivatives by similarity cluster prediction. J. Enzyme Inhib. Med. Chem..

[16] Ayoub L, Aissam E, Yassine K, Said E, Mohammed EM, Souad A (2018). A specific QSAR model for proteasome inhibitors from Oleaeuropaea and Ficuscarica. Bioinformation.

[17] Contrera JF, Maclaughlin P, Hall LH, Kier LB (2005). QSAR modeling of carcinogenic risk using discriminant analysis and topological molecular descriptors. Curr. Drug Discov. Technol..

[18] Konovalov DA, Llewellyn LE, Vander Heyden Y, Coomans D (2008). Robust cross-validation of linear regression QSAR models. J. Chem. Inf. Model..

[19] Cronin MTD, Basketter DA (2013). Multivariate QSAR analysis of a skin sensitization database. SAR QSAR Environ. Res..

[20] Papageorgiou SN (2020). Discriminant analysis: What it is and what is not. J. Orthod..

[21] He S (1978). Discriminant analysis. Crit. Rev. Clin. Lab. Sci..

[22] Landrum, G. A. *RDKit: Open-Source Cheminformatics Software, rdkit.Chem.Lipinski module[DB/CD]. Version 2021.03.1 ed*.

[23] Yang L, Wang Y, Chang J, Pan Y, Wei R, Li J, Wang H (2020). QSAR modeling the toxicity of pesticides against *Americamysis bahia*. Chemosphere.

[24] Petko A, Ivanka T, Pajeva Ilza (2015). Computational studies of free radical-scavenging properties of phenolic compounds. Curr. Top. Med. Chem..

[25] Bentes A, Borges R, Monteiro W, De Macedo L, Alves C (2011). Structure of dihydrochalcones and related derivatives and their scavenging and antioxidant activity against oxygen and nitrogen radical species. Molecules.

[26] Pandithavidana DR, Jayawardana SB (2019). Comparative study of antioxidant potential of selected dietary vitamins; computational insights. Molecules.

[27] Tanini D, Panzella L, Amorati R, Capperucci A, Pizzo E, Napolitano A, Menichetti S, D'Ischia M (2015). Resveratrol-based benzoselenophenes with an enhanced antioxidant and chain breaking capacity. Org. Biomol. Chem..

