# Supplementary material for: QSAR study of phenolic compounds and their anti-DPPH radical activity by discriminant analysis
Source: Sci Rep. 2022 May 12;12:7860. doi: 10.1038/s41598-022-11925-y (PMC9098848; doi:10.1038/s41598-022-11925-y)
Supplement: Supplementary file 1 — Supplementary Information. [file 41598_2022_11925_MOESM1_ESM.docx]

Table 1 The molecular structures and IC_50_ values of positive samples

| Mol. | | SMILE | IC_50_ | | Re.  No. |
| --- | --- | --- | --- | --- | --- |
| 1 | OC1=C(C)C=C(O)C(Br)=C1 | | 11.00 | | 1[1] |
| 2 | OC1=C(C)C=C(OC)C=C1 | | 17.00 | |  |
| 3 | CC1=C(O)C=CC(O)=C1 | | 12.00 | |  |
| 4 | OC1=C(CO)C=C(O)C=C1 | | 7.00 | |  |
| 5 | CC1=CC=C(C(C)CC(/C=C(C)/C)=O)C(O)=C1 | | 234.00 | | 2[2] |
| 6 | O=C(CC(O)CCC1=CC=C(O)C(OC)=C1)/C=C/C2=CC=C(O)C(OC)=C2 | | 29.00 | |  |
| 7 | O=C(C1=CC=C(O)C=C1)CC(O)CC(/C=C/C2=CC=C(O)C=C2)=O | | 39.00 | |  |
| 8 | O=C(CC(/C=C/C1=CC=C(O)C=C1)=O)/C=C/C2=CC=C(O)C=C2 | | 198.00 | |  |
| 9 | O=C(CC(/C=C/C1=CC=C(O)C(OC)=C1)=O)/C=C/C2=CC=C(O)C=C2 | | 47.00 | |  |
| 10 | O=C(CC(/C=C/C1=CC=C(O)C(OC)=C1)=O)/C=C/C2=CC(OC)=C(O)C=C2 | | 18.00 | |  |
| 11 | OC1=C(O)C=CC(C=O)=C1 | | 5.40 | | 3[3] |
| 12 | OC1=C(O)C(O)=CC(C=O)=C1 | | 6.00 | |  |
| 13 | OC1=C(O)C=CC(C=O)=C1O | | 11.60 | |  |
| 14 | OC1=CC(O)=C(C=O)C(O)=C1 | | 91.20 | |  |
| 15 | OC1=C(OC)C=C(C=O)C=C1 | | 97.60 | |  |
| 16 | NC1=C(C#N)N=CN1C2=CC=C(O)C=C2 | | 94.80 | |  |
| 17 | O=C(C1(C)CCC2=C(O1)C(C)=C(C)C(O)=C2C)O | | 9.50 | |  |
| 18 | OC1=C(C(C)(C)C)C=C(C)C=C1C(C)(C)C | | 72.74 | | 4[4] |
| 19 | OC1=C(C)C=C(O)C=C1O | | 35.08 | | 5[5] |
| 20 | CC1=CC(OC2=C(O)C=C(C)C=C2O)=C(O)C(O)=C1 | | 31.16 | |  |
| 21 | CC1=CC(OC2=CC(C)=CC(O)=C2)=C(O)C(O)=C1 | | 52.27 | |  |
| 22 | CC1=CC(OC2=CC(C)=CC(O)=C2OC3=C(O)C(O)=CC(C)=C3)=C(O)C(O)=C1 | | 21.22 | |  |
| 23 | CC1=CC(OC2=C(O)C=C(C)C=C2OC3=C(O)C=C(C)C=C3O)=C(O)C(O)=C1 | | 25.18 | |  |
| 24 | CC1=CC(OC2=CC(C)=CC=C2OC3=C(O)C=C(C)C=C3O)=C(O)C(O)=C1 | | 18.92 | |  |
| 25 | COC1=CC(/C=C/C(O)=O)=CC=C1O | | 45.30 | | 6[6] |
| 26 | COC1=CC(/C=C/C(NCCCCNC(/C=C/C2=CC=C(O)C=C2)=O)=O)=CC=C1O | | 70.40 | |  |
| 27 | COC1=CC(/C=C/C(NCCCCNC(/C=C/C2=CC=C(O)C(OC)=C2)=O)=O)=CC=C1O | | 38.50 | |  |
| 28 | OC1=CC(/C=C/C(O)=O)=CC=C1O | | 13.80 | | 7[7] |
| 29 | OC1=CC=C(O)C=C1OC | | 18.56 | | 8[8] |
| 30 | OC1=CC=C(O)C=C1 | | 28.75 | |  |
| 31 | CC1=CC(O)=CC(C)=C1 | | 173.44 | |  |
| 32 | C/C(C)=C\CC1=CC(O)=C(OC)C=C1O | | 23.68 | |  |
| 33 | C/C(C)=C\CC1=C(O)C=C(O)C=C1 | | 81.22 | |  |
| 34 | C/C(C)=C\CC1=CC(O)=CC=C1O | | 18.68 | |  |
| 35 | C/C(C)=C\CC1=C(C)C=C(O)C=C1O | | 60.34 | |  |
| 36 | C/C(C)=C\CC1=CC(O)=C(O)C=C1 | | 21.38 | |  |
| 37 | C/C(C)=C\CC1=C(O)C=C(O)C=C1O | | 114.54 | |  |
| 38 | C/C(C)=C\CC1=C(O)C=C(O)C(C/C=C(C)\C)=C1 | | 50.94 | |  |
| 39 | C/C(C)=C\CC1=C(O)C=C(C/C=C(C)\C)C(O)=C1 | | 21.19 | |  |
| 40 | C/C(C)=C\CC1=C(O)C=C(O)C(C/C=C(C)\C)=C1C | | 48.32 | |  |
| 41 | C/C(C)=C\CC1=C(C/C=C(C)\C)C=C(O)C(O)=C1 | | 19.06 | |  |
| 42 | C/C(C)=C\CC1=C(O)C(C/C=C(C)\C)=C(O)C=C1O | | 45.36 | |  |
| 43 | CC1(C)CCC2=C(O1)C=CC(O)=C2 | | 35.63 | |  |
| 44 | O=C(OC1=CC=C(C(C)(C)C)C=C1)/C=C/C2=CC=C(O)C(OC)=C2 | | 150.00 | | 9[9] |
| 45 | O=C(OC1=CC=C(C(C)C)C=C1)/C=C/C2=CC=C(O)C(OC)=C2 | | 130.00 | |  |
| 46 | O=C(OC1=CC=C(Cl)C(C)=C1)/C=C/C2=CC=C(O)C(OC)=C2 | | 130.00 | |  |
| 47 | O=C(OC1=CC=C(OC)C=C1)/C=C/C2=CC=C(O)C(OC)=C2 | | 140.00 | |  |
| 48 | O=C(OC1=CC=C(NC(C)=O)C=C1)/C=C/C2=CC=C(O)C(OC)=C2 | | 140.00 | |  |
| 49 | OC1=CC=C(O)C=C1C(C)(C)C | | 5.00 | | 10[10] |
| 50 | OC1(C(O)=O)CC(O)C(O)C(OOC(C2=CC(O)=C(O)C(O)=C2)=O)C1 | | 18.70 | | 11[11] |
| 51 | OC1(C(O)=O)CC(OOC(C2=CC(O)=C(O)C(O)=C2)=O)C(O)C(OOC(C3=CC(O)=C(O)C(O)=C3)=O)C1 | | 7.10 | |  |
| 52 | OC1(C(O)=O)CC(OOC(C2=CC(O)=C(O)C(O)=C2)=O)C(OOC(C3=CC(O)=C(O)C(O)=C3)=O)C(OOC(C4=CC(O)=C(O)C(O)=C4)=O)C1 | | 3.90 | |  |
| 53 | OC1=CC(/C=C/C(O[C@@H]2C[C@](O)(C(O)=O)C[C@@H](O)[C@H]2O)=O)=CC=C1O | | 22.80 | | 12[12] |
| 54 | COC1=CC(/C=C/C(O)=O)=CC(OC)=C1O | | 32.10 | |  |
| 55 | OC1=CC=CC=C1N | | 15.10 | |  |
| 56 | OC1=CC=CC(N)=C1 | | 18.80 | |  |
| 57 | NCCC1=CC=C(O)C(O)=C1 | | 9.50 | |  |
| 58 | O=C1C2=C(O)C(O)=C(OC)C=C2[C@](O[C@@H](CCC)C3)([H])[C@]3([H])O1 | | 23.40 | | 13[13] |
| 59 | O=C1C2=C(O)C(O)=C(OC)C=C2C[C@@H](C[C@@H](O)CCC)O1 | | 16.40 | |  |
| 60 | O=C1OC(C2=CC=C(O)C=C2)CC3=C1C(O)=CC(OC)=C3 | | 33.30 | | 14[14] |
| 61 | OC1=CC(OC2=C(O)C=C3C(OC(C=C(OC4=C(O)C=C(O)C=C4O)C(O)=C5O)=C5O3)=C2O)=CC(O)=C1 | | 3.41 | | 15[15] |
| 62 | OC1=C(C(C)(C)C)C=C(SC(C)(C)SC2=CC(C(C)(C)C)=C(O)C(C(C)(C)C)=C2)C=C1C(C)(C)C | | 9.30 | | 16[16] |
| 63 | OC1=C(O)C=C([C@@H](C2=CC=C(O)C(O)=C2)C(C)C3CC)C3=C1 | | 2.80 | | 17[17] |
| 64 | O=C(OCC1=CC=C(O[C@H]2[C@@H](O)[C@@H](O)C(O)[C@H](CO)O2)C(O)=C1)C3=CC(O[C@@H]4[C@H](O)[C@H](O)C(O)[C@@H](CO)O4)=C(O)C=C3 | | 214.81 | | 18[18] |
| 65 | O=C(OCC1=CC=C(O[C@H]2[C@@H](O)[C@@H](O)C(O)[C@H](CO)O2)C(O)=C1)C3=CC=C(O)C=C3 | | 293.73 | |  |
| 66 | OC1=CC=C(C(O)=O)C=C1OC2O[C@H](CO)[C@@H](O)[C@H](O)[C@H]2O | | 279.36 | |  |
| 67 | OC1=C(CCCCCCC)C(C=O)=C(O)C(C/C=C(C)/C)=C1 | | 37.12 | | 19[19] |
| 68 | OC1=C(CC/C=C/C=C/C)C(C=O)=C(O)C(C/C=C(C)/C)=C1 | | 38.28 | |  |
| 69 | OC1=CC=C(O)C(CO)=C1CC/C=C/C=C/C | | 9.30 | | 20[20] |
| 70 | OC1=CC=C(O)C(CO)=C1CC/C=C/C=C/C | | 17.60 | |  |
| 71 | OC1=C(OC(/C=C/CCC)=C2)C2=C(C=O)C(O)=C1C/C=C(C)/C | | 24.20 | |  |
| 72 | OC1=C(OC(/C=C/C=C/C)=C2)C2=C(C=O)C(O)=C1C/C=C(C)/C | | 20.90 | |  |
| 73 | OC1=CC(C/C=C(C)/C)=C(O)C(C=O)=C1/C=C/C=C/CCC | | 9.80 | |  |
| 74 | OC1=CC(C/C=C(C)/C)=C(O)C(C=O)=C1/C=C/CCCCC | | 11.20 | |  |
| 75 | OC1=CC(C/C=C(C)/C)=C(O)C(C=O)=C1CC/C=C/C=C/C | | 16.30 | |  |
| 76 | OC1=CC(C/C=C(C)/C)=C(O)C(C=O)=C1/C=C/C=C/C=C/C | | 7.60 | |  |
| 77 | O=C(NCCCCNC(/C=C/C1=CC=C(O)C(OC)=C1)=O)C2=CC=C(O)C=C2 | | 63.00 | | 21[21] |
| 78 | O=C(OC(C(OC)=O)CC1=CC=C(OC2=CC=C(O)C=C2)C(O)=C1)/C=C/C3=CC=C(O)C(O)=C3 | | 56.00 | |  |
| 79 | OC1=C(O)C=CC(CCOC([C@@H](O)C)=O)=C1 | | 39.87 | | 22[22] |
| 80 | OC1=C(O)C=CC(CCOC(CCC(OCCC2=CC=C(O)C(O)=C2)=O)=O)=C1 | | 31.53 | |  |
| 81 | O=C([C@H](C)OC1=CC=C(CC(O)=O)C=C1O)OCCC2=CC(O)=C(O)C=C2 | | 24.15 | |  |
| 82 | OC1=C(O)C=CC(CCOC(CCC(O)=O)=O)=C1 | | 46.41 | |  |
| 83 | OC(C(O)=C1)=CC=C1C2=CN=C(C)C=C2 | | 38.12 | |  |
| 84 | OC1=CC(C2=CC(C3=CC(O)=C(O)C=C3)=CN=C2)=CC=C1O | | 40.16 | |  |
| 85 | O=C(O)CC1=CC(O)=C(O)C=C1 | | 6.07 | |  |
| 86 | O=C(OCCC1=CC=C(O)C(O)=C1)CC2=CC(O)=C(O)C=C2 | | 19.22 | |  |
| 87 | OC1=C(O)C=C(C2=CC(O)=C(O)C=C2)C=C1 | | 54.08 | |  |
| 88 | OC1=CC2=C(C(OCC2)C3=CC=C(O)C(O)=C3)C=C1O | | 33.14 | |  |
| 89 | OC1=C(O)C(O)=CC(CC(NC2=CC=CC=C2)=O)=C1 | | 6.00 | | 23[23] |
| 90 | OC1=C(O)C(O)=CC(CC(NCC2=CC=C(O)C=C2)=O)=C1 | | 10.50 | |  |
| 91 | OC1=C(O)C(O)=CC(CC(NCC2=CC=C(O)C(O)=C2)=O)=C1 | | 31.80 | |  |
| 92 | OC1=C(O)C(O)=CC(CC(NCCC2=CC(Cl)=CC=C2)=O)=C1 | | 9.60 | |  |
| 93 | OC1=C(O)C(O)=CC(CC(NCCC2=CC=C(O)C=C2)=O)=C1 | | 6.70 | |  |
| 94 | OC1=C(O)C(O)=CC(CC(NCCC2=CC=C(O)C(O)=C2)=O)=C1 | | 3.80 | |  |
| 95 | OC1=C(O)C(O)=CC(CC(NCCCC2=CC=CC=C2)=O)=C1 | | 7.10 | |  |
| 96 | OC1=C(OC)C=C(O[C@@H]2O[C@H](CO)[C@@H](O)[C@H](O)[C@H]2O)C=C1 | | 27.60 | | 24[24] |
| 97 | OC1=CC(/C=C/C(OC(C(O)=O)CC2=CC=C(O)C(O)=C2)=O)=CC=C1O | | 12.70 | |  |
| 98 | CC(C)C(C(O)=C1O)=CC2=C1[C@]34[C@](CC2)([H])[C@@](C)(C(OC4=O)O)CCC3 | | 24.80 | | 25[25] |
| 99 | CC(C)C(C(O)=C1O)=CC2=C1[C@]34[C@](CC2)([H])[C@](COC4=O)(C)CCC3 | | | 29.90 |  |

Table 2 The molecular structures of negative samples

| Mol. | SMILE |
| --- | --- |
| 1 | CC(C)C1=C(O)C(C(C)C)=CC=C1 |
| 2 | OC(CNC)C1=CC=C(O)C(OC)=C1 |
| 3 | BrCCC1=CC=C(O)C=C1 |
| 4 | OC1=C(CC2=CC=C(Cl)C=C2)C=C(CC)C=C1 |
| 5 | OC1=C(CC)C(C)=CC(C)=C1CC |
| 6 | OC1=CC([C@]2([H])CN(CCC)C[C@@](CSC)([H])C2)=CC=C1 |
| 7 | OC1=CC=C(C(C)CC)C=C1 |
| 8 | CC(CC)C1=C(O)C(C(C)CC)=CC(C)=C1 |
| 9 | CC(C)(C)C1=C(O)C(C)=CC=C1 |
| 10 | CC(CC)C1=C(O)C(CCCC)=CC=C1 |
| 11 | CC(C)C1=C(O)C(C(C)C)=C(C)C=C1C |
| 12 | OC1=CC=C(C(O)C(NC)C)C=C1 |
| 13 | OC1=CC(CCNCCC)=CC=C1 |
| 14 | OC1=CC=C(CCNOC)C=C1 |
| 15 | CCC1=CC=C(N2CCC[C@@H](C3=CC=CC(O)=C3)C2)C=C1 |
| 16 | CN1CCC[C@@H](C2=CC=CC(O)=C2)C1 |
| 17 | OC1=CC([C@@H](C2)CCCN2CCCC)=CC=C1 |
| 18 | CC(C)C1=C(O)C(C(C)C)=CC(I)=C1 |
| 19 | COC1=CC(CC2=CN=C(N)N=C2N)=CC=C1O |
| 20 | OC1=CC=CC(CCCl)=C1 |
| 21 | OC1=CC(CC)=CC=C1 |
| 22 | OC1=CC=CC([C@H](CCC2)CN2CCC3=CC=CC=C3)=C1 |
| 23 | CC1=CC(CCC2=CC=C(F)C=C2)=CC(C)=C1O |
| 24 | OC1=C(C(C)CCCC)C=CC=C1CCC |
| 25 | OC1=CC=CC=C1C(C)CC |
| 26 | OC1=CC=CC([C@@H](CCC2)CN2CCC3=CC=CC=C3)=C1 |
| 27 | CC1=C(O)C(C)=CC=C1CC2=NCCN2 |
| 28 | OC1=CC=C(CC=C)C=C1 |
| 29 | OC1=C(C2CCCC2)C=CC=C1 |
| 30 | OC1=CC=C(CCNCCCCCC2=CC=CC=C2)C=C1 |
| 31 | OC1=CC=C(CCCC)C=C1 |
| 32 | OC1=CC=C(CCCCCCCCC)C=C1 |
| 33 | OC1=CC=CC(C2(CCC)CCN(C)C2)=C1 |
| 34 | OC1=C(CCN(C)C)C=C(OC)C=C1 |
| 35 | OC1=CC(CC(C)N)=CC=C1OC |
| 36 | OC(C=C1)=CC=C1CC(CC2)CCN2CCCC3=CC=CC=C3 |
| 37 | OC1=C(CC(C)N)C=C(OC)C=C1 |
| 38 | OC1=CC=C(C2CN(CCCCC3=CC=CC=C3)CC2)C=C1 |
| 39 | OC1=C(CC(C)N(C)C)C=C(OC)C=C1 |
| 40 | OC1=CC=C(CCN(CCC)CCC)C=C1 |
| 41 | OC1=CC=C(C(O)CC)C=C1 |
| 42 | CC(C1=CC=CC=C1)(C)C2=CC=C(O)C=C2 |
| 43 | OC1=CC=CC([C@@H]2OCCN(CCC)C2)=C1 |
| 44 | OC1=C(C/C=C(C)/C)C=C(C(C)(C=C)C)C=C1 |
| 45 | OC1=CC=C(C(O)CCCCC)C=C1 |
| 46 | OC1=CC=CC=C1CCN2CCCC2 |
| 47 | CCCN(CCC1)CC1C2=CC(O)=CC=C2 |
| 48 | CC(C)C1=C(O)C(C)=CC=C1 |
| 49 | CC(C)C1=CC=CC(C2CCCCC2)=C1O |
| 50 | CC(CCC)C1=CC=CC=C1O |
| 51 | CC1=CC(CCC2=CC=CS2)=CC(C)=C1O |
| 52 | CCCN(CCC1)CC1C2=CC=C(O)C=C2 |
| 53 | OC/C=C/C1=CC=CC=C1O |
| 54 | OC1=CC=CC=C1C(CN(C)C)C2(O)CCCCC2 |
| 55 | OC(CN)C1=CC=C(C)C(O)=C1 |
| 56 | OC1=CC=C(N(CCCl)CCCl)C(F)=C1 |
| 57 | CC(C)(C)C1=C(O)C(C(C)(C)C)=CC(COC)=C1 |
| 58 | OC1=CC=CC=C1CCN2CCC[C@H]2C |
| 59 | OC1=C(OC2=CC=CN=C2)C=CC(CCCCCC)=C1 |
| 60 | OC1=CC([C@]2(OC)CCCC[C@H]2CNC)=CC=C1 |
| 61 | CC1=CC=C(CCN(CCC)CCC)C=C1O |
| 62 | CC1=CC(C(C)(C)C)=CC=C1O |
| 63 | OC1=CC=CC(C(O)CCCC)=C1 |
| 64 | OC1=C(CCC2=CC=C(OC)C(OC)=C2)C=CC=C1 |
| 65 | OC1=CC=CC=C1CCC2=NCCN2 |
| 66 | COC1=CC(CN(CC)CC)=CC(OC)=C1O |
| 67 | NCC1=C(O)C(C)=CC(CCCC)=C1 |
| 68 | NCC1=C(O)C(CCC)=CC(CCC)=C1 |
| 69 | NCC1=C(O)C(C)=CC(C(C)(C)C)=C1 |
| 70 | OC1=CC=CC(C2CN(CCC)CCC2)=C1 |
| 71 | OC1=CC(C2CN(CCC)CC2)=CC=C1 |
| 72 | OC1=CC(C2=NN=C(NN)S2)=CC=C1 |
| 73 | OC1=C(C2CCCC=C2)C=CC=C1 |
| 74 | OC1=CC=C(CCNC(N)=N)C=C1 |
| 75 | OC(C=C1)=CC=C1CCCCN(CC2)CCC2C3=CC=CC=C3 |
| 76 | OC1=C(CC2=CC=C(C)C=C2)C=CC=C1 |
| 77 | OC1=CC=C(C[C@@H](NC)[C@H](O)CC/C=C/CCCCC)C=C1 |
| 78 | OC1=C([C@@H](C)CC)C=CC=C1[C@H](C)CC |
| 79 | CC(C1=CC=CC=C1)(C)C2=C(O)C=CC(C)=C2 |
| 80 | CC(C)(C)C1=CC(C(C)C2=CC=CC=C2)=C(O)C=C1 |
| 81 | CC(C)(C)C1=C(O)C(C(C)(C)C)=CC(C(C)(C)CO)=C1 |
| 82 | OC1=CC=CC(OCCN(C)C)=C1 |
| 83 | OC1=CC=C(C[C@H](C)NC)C=C1 |
| 84 | OC1=CC=C(C[C@@H](CO)NCCC)C=C1 |
| 85 | OC1=CC=CC=C1CN2CCCCC2 |
| 86 | OC1=C(C)C(CCCCNCCC)=C(C)C=C1C |
| 87 | OC1=CC=CC(C2(C)CCN(C)CC2)=C1 |
| 88 | OC1=CC(C(C2COCOC2)N(C)C)=CC=C1 |
| 89 | OC1=CC=C(CCC2=CC(C)=NO2)C=C1 |
| 90 | OC1=C(OCCCO)C=CC=C1C(CN)O |
| 91 | OC1=CC(CCCC2=CC=C(OC)C=C2)=CC=C1 |
| 92 | OC1=CC(CCCC2=CC=CC=C2)=CC=C1CN(C)C |
| 93 | OC1=CC=C(CC2=CNC(N)=N2)C=C1 |
| 94 | OC1=CC=C(CNCCCC)C=C1 |
| 95 | OC1=CC(CCCCC)=C(C)C=C1 |
| 96 | CC1=CC(C)=C(NC2=CC=CC(O)=C2)C(C)=C1 |
| 97 | OC1=CC=CC=C1CCNCCC |
| 98 | OC1=CC=CC=C1CCN(CCC)CCC |
| 99 | COC1=CC(OC)=CC=C1CCCC2=CC=CC(O)=C2 |
| 100 | OC1=C([C@H](C)CC)C=CC=C1[C@H](C)CC |
| 101 | OC1=CC=C(CCCC2=CC=CC=C2)C=C1 |
| 102 | OC1=CC=C(C2CCC(C)CC2)C=C1 |
| 103 | O[C@H]1C(O[C@H]2[C@]3([H])[C@](O)(C)[C@@H](OC(/C=C/C4=CC(O)=  C(OC)C=C4)=O)C[C@]3(O)C(C(OC)=O)=CO2)OC(CO)[C@@H](O)[C@@H]1O |
| 104 | O[C@H]1C(O[C@H]2[C@]3([H])[C@](O)(C)[C@@H](OC(/C=C/C4=CC=C(O)  C=C4)=O)C[C@]3(O)C(C(OC)=O)=CO2)OC(CO)[C@@H](O)[C@@H]1O |
| 105 | O[C@H]1C(O[C@H]2[C@]3([H])C(CO)=CC[C@]3([H])C(C(OC)=O)=CO2)OC  (OC(/C=C/C4=CC(O)=C(OC)C=C4)=O)[C@@H](O)[C@@H]1O |

**References:**

[1]. Leutou, A.S., New Production of 5-Bromotoluhydroquinone and 4-O-Methyltoluhydroquinone from the Marine-Derived Fungus Dothideomycete sp. Journal of Microbiology and Biotechnology, 2012. 22(1): p. 80-83.

[2]. Akter, J., et al., Antioxidant activity of different species and varieties of turmeric (Curcuma spp): Isolation of active compounds. Comparative Biochemistry and Physiology Part C: Toxicology & Pharmacology, 2019. 215: p. 9-17.

[3]. Correia, C., et al., Synthesis and radical scavenging activity of phenol-imidazole conjugates. Bioorganic & Medicinal Chemistry Letters, 2014. 24(12): p. 2768-2772.

[4]. Pongkittiphan, V., W. Chavasiri and R. Supabphol, Antioxidant Effect of Berberine and its Phenolic Derivatives Against Human Fibrosarcoma Cells. Asian Pacific Journal of Cancer Prevention, 2015. 16(13): p. 5371-5376.

[5]. Pongkittiphan, V., C. Warinthorn and S. Roongtawan, Antioxidant Effect of Berberine and its Phenolic Derivatives

Against Human Fibrosarcoma Cells. Asian Pac J Cancer Prev, 2015. 16(13): p. 5371-6.

[6]. Choi, S.W., et al., Antioxidant and Antimelanogenic Activities of Polyamine Conjugates from Corn Bran and Related Hydroxycinnamic Acids. Journal of Agricultural and Food Chemistry, 2007. 55(10): p. 3920-3925.

[7]. Mahakunakorn, P., et al., Antioxidant and free radical-scavenging activity of Choto-san and its related constituents. Biol Pharm Bull, 2004. 27(1): p. 38-46.

[8]. Osorio, M., et al., Synthesis and DPPH Radical Scavenging Activity of Prenylated Phenol Derivatives. Molecules, 2012. 17(1): p. 556-570.

[9]. Ergün, B.Ç., et al., Synthesis, antioxidant and antimicrobial evaluation of simple aromatic esters of ferulic acid. Archives of Pharmacal Research, 2011. 34(8): p. 1251-1261.

[10]. Badary, O.A., et al., Thymoquinone Is a Potent Superoxide Anion Scavenger. Drug and Chemical Toxicology, 2003. 26(2): p. 87-98.

[11]. Ijaz, F., et al., Antioxidative iridoid glycosides from the sky flower (Duranta repens Linn). Journal of Enzyme Inhibition and Medicinal Chemistry, 2011. 26(1): p. 88-92.

[12]. Cos, P., et al., In Vitro Antioxidant Profile of Phenolic Acid Derivatives. Free Radical Research, 2009. 36(6): p. 711-716.

[13]. Tianpanich, K., et al., Radical Scavenging and Antioxidant Activities of Isocoumarins and a Phthalide from the Endophytic FungusColletotrichum sp. Journal of Natural Products, 2011. 74(1): p. 79-81.

[14]. Wu, J., et al., Antioxidants and a new dihydroisocoumarins from Polygala hongkongensis Hemsl. 2015. 21(7): p. 580-584.

[15]. Heo, S., et al., Identification of chemical structure and free radical scavenging activity of diphlorethohydroxycarmalol isolated from a brown alga, Ishige okamurae. Journal of microbiology and biotechnology, 2008. 18(4): p. 676.

[16]. Ko, F.N., et al., Isoorientin-6"-O-glucoside, a water-soluble antioxidant isolated from Gentiana arisanensis. Biochim Biophys Acta, 1998. 1389(2): p. 81-90.

[17]. Feng-Nien Ko, C.L.Y.K., Antioxidant properties of demethyldiisoeugenol. Biochimica et Biophysica Atca, 1995: p. 145-152.

[18]. Nassar, M.I., et al., Phenolic metabolites from Pyrus calleryana and evaluation of its free radical scavenging activity. Carbohydrate research, 2011. 346(1): p. 64-67.

[19]. Li, Y., et al., A New Radical Scavenging Anthracene Glycoside, Asperflavin Ribofuranoside, and Polyketides from a Marine Isolate of the Fungus Microsporum. Chemical & pharmaceutical bulletin, 2006. 54(6): p. 882-883.

[20]. Sun, S., et al., Three new polyketides from marine-derived fungus Aspergillus glaucus HB1-19. Journal of asian natural products research, 2013. 15(9): p. 956-961.

[21]. Choudhary, M.I., et al., Two New Antioxidant Phenylpropanoids from Lindelofia stylosa. Chemistry & biodiversity, 2008. 5(12): p. 2676-2683.

[22]. Xiao, H., et al., Five New Phenolic Compounds with Antioxidant Activities from the Medicinal Insect Blaps rynchopetera. Molecules, 2017. 22(8): p. 1301.

[23]. Kim, J., V.S. Hong and J. Lee, Antioxidant activity of 3,4,5-trihydroxyphenylacetamide derivatives. Archives of Pharmacal Research, 2014. 37(3): p. 324-331.

[24]. Tadrent, W., et al., A new sulfonylated flavonoid and other bioactive compounds isolated from the aerial parts of Cotula anthemoides L. Natural product research, 2017. 31(12): p. 1437-1445.

[25]. Bustos-Brito, C., et al., Structure and Absolute Configuration of Abietane Diterpenoids fromSalvia clinopodioides : Antioxidant, Antiprotozoal, and Antipropulsive Activities. Journal of Natural Products, 2019. 82(5): p. 1207-1216.
